# Supplementary material for: A UMLS-based spell checker for natural language processing in vaccine safety
Source: BMC Med Inform Decis Mak. 2007 Feb 12;7:3. doi: 10.1186/1472-6947-7-3 (PMC1805499; doi:10.1186/1472-6947-7-3)
Supplement: Additional file 13 — RAP application directory. Sets up directory for RAP (RDF application for PHP) source code [file 1472-6947-7-3-S13.gz › rap/doc/tutorial/grddl_parser.htm]

Tutorial: GRDDL Parser


### RDF API for PHP V0.9.1

# Tutorial: GRDDL Parser

This turorial is part of the RAP - Rdf API for PHP
documentation.

The RAP GRDDL parser allows you to extract RDF-data from XHTML documents.

The GRDDL parser requires PHP 5.1 or later, because it uses PHP5's build in
xslt-processor.

There a several approaches to extract RDF-data from XHTML documents and there
is a W3C
best-practice "RDF in XHTML Taskforce" looking at the issue. RAP
provides an implementation of the GRDDL proposal, currently discussed by the
working group. GRDDL stands for "Gleaning
Resource Description from Dialects of Languages" and uses XSLT to extract
RDF-data from XHTML.

(http://www.w3.org/TR/grddl/)

### 1. XHTML Input Document

The Input document has to contain at least one reference to an XSLT stylesheet
which describes how your rdf-data is encoded in the XHTML document. For example,
this document

 ```` ```
<html xmlns="http://www.w3.org/1999/xhtml">
  <head profile="http://www.w3.org/2003/g/data-view">
    <title>Some Document</title>

    <link rel="transformation"
       href="http://www.w3.org/2000/06/dc-extract/dc-extract.xsl" />
    <meta name="DC.Subject"
       content="ADAM; Simple Search; Index+; prototype" />
    ...
  </head>
  ...
</html>
``` ````

refers to the dc-extract.xsl stylesheet and contains the following
RDF statements:  
 ```` ```
<rdf:RDF
  xmlns:dc="http://purl.org/dc/elements/1.1/"
  xmlns:rdf="http://www.w3.org/1999/02/22-rdf-syntax-ns#"
  >
  <rdf:Description rdf:about="">
    <dc:subject>ADAM; Simple Search; Index+; prototype</dc:subject>
  </rdf:Description>
</rdf:RDF>
``` ```` 

The stylesheet is located at http://www.w3.org/2000/06/dc-extract/dc-extract.xsl

### 2. Using RAP's GRDDL Parser

To parse an rdf-data containing xhtml document, simply use the `load()`
function of any RAP model. If the file extension of your XHTML
file is ".html" or".htm" then RAP uses the GRDDL parser
automatically. Otherwise you have to explicitly specifiy the parser in the load
call `$model->load('doc.html', "GRDDL");`

`define("RDFAPI_INCLUDE_DIR", "C:/Apache/htdocs/rdf_api/api/");   
include(RDFAPI_INCLUDE_DIR . "RdfAPI.php");`

`$model = new MemModel();  
$model->load('doc.html');`

### 3. Further Information about GRDDL

For further information and examples see

- Gleaning Resource Descriptions from
  Dialects of Languages (GRDDL)
- GRDDL Data Views: Getting Started,
  Learning More
- RDF
  in XHTML Taskforce

Some Stylesheets for commonly known vocabularies are:

- GeoURL markup and grokGeoURL.xsl
- RDF Site Summaries in
  XHTML, using home2rss.xsl
